# Understanding Patient and Physician Perspectives on Exclusive Enteral Nutrition in Adults with Crohn’s Disease: Bridging the Gap in Nutritional Therapy

**DOI:** 10.3390/nu17182945

**Published:** 2025-09-12

**Authors:** Ramit Magen-Rimon, David Mamet, Meis Assaf, Zohar Tal-Leshinsky, Oxana Libman, Matti Waterman, Haggai Bar-Yoseph

**Affiliations:** 1 Pediatric Gastroenterology Institute, Rambam Health Care Campus, Haifa 3109601, Israel; 2 The Ruth & Bruce Rappaport Faculty of Medicine, Technion-Israel Institute of Technology, Haifa 3200003, Israel; mamet.david24@gmail.com (D.M.); meisassaf05@gmail.com (M.A.); z_talleshinsky@rambam.health.gov.il (Z.T.-L.); m_waterman@rmc.gov.il (M.W.); h_bar-yoseph@rmc.gov.il (H.B.-Y.); 3 Department of Gastroenterology, Rambam Health Care Campus, Haifa 3109601, Israel; o_libman@rmc.gov.il

**Keywords:** Crohn’s disease, exclusive enteral nutrition, attitude survey, acceptance

## Abstract

**Background and Aims:** Exclusive enteral nutrition (EEN) effectively induces remission in pediatric Crohn’s disease (CD), but reasons for its limited use in adults remain unclear. This study aimed to identify and compare patients’ and physicians’ perspectives on barriers to EEN use, in order to inform strategies to enhance its utilization. **Methods:** An online questionnaire was distributed to adult CD patients and gastroenterologists, collecting data on demographics, disease characteristics, previous EEN exposure, and attitudes toward EEN and potential barriers (Likert scale 1–5). Comparative analysis and logistic regression identified factors associated with reluctance toward EEN. **Results:** A total of 315 CD patients (mean age 36.7) and 42 physicians completed the survey. Previous EEN use was reported by 40%, while 20.3% of the entire cohort were reluctant to use it. The only factor that predicted reluctance was current use of advanced therapy (OR 2.06 [95%CI 1.05–4.35]). Among physicians, 71% had prescribed EEN, and 81% were willing to do so. Key barriers were lack of food variety (65% Likert score > 3) and reduced social interaction (59%) among patients and social interaction (67.3%) and taste concerns (54.7%) among physicians. Patients rated hunger sensation and lack of food variety concerns as more significant barriers than physicians. Patients identified direct communication with staff (68.6% Likert score > 3) and full cost coverage (65%) as facilitators for adherence. Notably, 87% wanted more information from their doctors. **Conclusions:** Most adult CD patients and physicians are open to discuss EEN. Removing barriers related to palatability and diversity of enteral nutrition, as well as shortening of EEN duration, may enhance acceptance of and adherence to EEN.

## 1. Introduction

Crohn’s disease (CD) is a chronic disease and relapsing–remitting inflammatory condition that can affect any part of the intestinal tract and often requires acute treatment to induce remission, particularly during disease exacerbation. In adult patients, corticosteroids are commonly used for this purpose, despite their well-documented adverse effects [1], highlighting the need for a safer and equally agreed-upon alternative. Increasing evidence suggests that dietary patterns play a major role in disease pathogenesis and exacerbations through influencing the intestinal microbiome composition, negatively affecting mucosal barrier function and immune response [2]. This has led to the development of several dietary interventions for CD treatment in recent decades [3]. Among them, exclusive enteral nutrition (EEN) is the most studied diet and has the strongest evidence for its efficacy in inducing remission in patients with CD. EEN involves the ingestion of a liquid formula as an exclusive diet for several weeks with no solid food intake allowed [3]. It has been demonstrated to be superior to oral corticosteroids in achieving mucosal healing with fewer side effects and is therefore the preferred induction treatment for pediatric CD according to the European guidelines [3,4,5,6]. In adult CD patients, the European Crohn’s and Colitis Organization (ECCO) guidelines suggest EEN for induction of remission in patients with mild–moderate disease who wish to avoid corticosteroids [7,8]. While mostly prescribed for mild–moderate disease, EEN has demonstrated efficacy in inducing clinical, chemical, and endoscopic remission in complicated disease as well (i.e., inflammatory strictures, abdominal abscesses, and intestinal fistulae) [7,8,9,10] and has the added benefit of improving nutritional status. The exact mechanism by which EEN induces remission is not fully understood. However, several theories have been proposed: exclusion of dietary antigens, modulation of the intestinal microbiome, a direct anti-inflammatory effect, and improvement in mucosal barrier function [11]. Despite these clear advantages, EEN remains underutilized in adult CD patients [11,12]. The reasons for this discrepancy are not fully understood. Reported barriers include low palatability, missing the sensation of eating, and the social limitations of a restricted diet [13]. However, patients report that these issues become more manageable after the first 1–2 weeks, as symptoms improve [11].

In addition to patient barriers, physician-related factors may also contribute to EEN’s underutilization. These may include lack of familiarity with prescribing EEN and inadequate dietitian support. Some clinicians may hold the perception that EEN would be unacceptable to adult patients, leading to its exclusion as a treatment option during shared decision-making discussions.

Accumulating evidence supports the notion that EEN could be a valuable underexploited tool in adult CD management [7,8,9]. To optimize the use of EEN in adult care, a deeper understanding of the perceived barriers among both patients and physicians is needed. Identifying and addressing the barriers could lead to more robust adoption of this safe and effective therapy worldwide. This study therefore aimed to explore the perspectives of patients with CD and gastroenterologists regarding the use of EEN, to identify factors limiting EEN utilization, and to highlight potential strategies to enhance implementation and adherence in the adult CD population.

## 2. Methods

This was a cross-sectional study involving CD patients aged 18 and older and gastroenterologists who treat patients with inflammatory bowel disease (IBD). Online, fully anonymous questionnaires were developed with expert consultation and validated [14] for both patients and physicians [The English translated questionnaires appear in Files S1 and S2, respectively. Translation from Hebrew was performed by a staff member fluent in both languages (DM)]. In the process of validation, the questionnaires were distributed to 8 pediatric gastroenterologists who scored the items according to their relevance on a 1–4 scale (1—irrelevant; 4—most relevant). Each item included in the questionnaire achieved a content validity index greater than 0.8, indicating that over 80% of the experts rated the item as relevant. Items meeting this threshold were considered valid for inclusion. The patient questionnaire was distributed to patients treated at Rambam Health Care Campus, Haifa, Israel, as well as on social networks. The physician questionnaire was distributed among referral/community gastroenterologists from 14 medical centers as well as from the Health Maintenance Organizations in Israel. Both patients and physicians had access to the questionnaires via QR code. The study was approved by the local Institutional Review Board at Rambam Health Care Campus (No. 0387-23-RMB) on 5 February 2024.

The patient questionnaire covered demographics, disease history, current treatment, knowledge of EEN, past experience with the diet, perceived barriers, and potential support factors. The respondents were also given the option to provide additional comments in an open-text section (File S1). The physician questionnaire covered demographics, experience with IBD and EEN, perception of EEN’s efficacy, potential patient and physician barriers to recommending EEN, and potential support factors (File S2). The respondents were asked to score the potential barriers and the support factors on a Likert scale of 1–5 (1 represented disagreement with the statement, while 5 represented full agreement).

### Statistical Analysis

Descriptive statistics with continuous variables are presented as the mean ± standard deviation (SD) or median and range according to distribution, and categorical variables are reported as frequencies and percentages. To better understand the effect of baseline variables, barriers, and supporting factors on EEN receptiveness, patients were grouped by their responses, with ‘Yes’ and ‘Maybe’ responses combined to ‘willingness to try EEN’ and negative responses classified as ‘EEN reluctance’. All subsequent analyses were stratified accordingly. First, we compared baseline factors between the groups using Chi-square tests or t-tests according to data type for both patients and physicians. We then performed multivariate logistic regression to identify factors associated with patient reluctance toward EEN and physician willingness to offer it. The multivariate models for patients included the following variables: age, sex, ethnicity, marital status, >12 years of education, age at CD diagnosis, previous hospitalization for CD, history of CD-related surgery, active CD, past experience with EEN, perceived positive response to EEN, currently receiving corticosteroids, currently receiving immunomodulator therapy, and currently receiving advanced therapy. For physicians, the following variables were included: age, sex, years practicing gastroenterology, >15 years IBD care experience, treating >60 IBD patients per year, pediatric gastro collaboration, previous EEN prescription, and addressing nutrition during most visits. To investigate the differences regarding perceived barriers among patients and physicians, their responses were compared using a Mann–Whitney U test. Lastly, an association network was generated to visualize how different barriers contribute to reluctance to try EEN. The network was created using Kendall’s Tau test for statistical association: relationships between barriers and EEN reluctance are represented as nodes, while the correlation between barriers is represented as edges. Node size represents Kendall’s Tau correlation coefficient scaled by 100, while edge strength is defined as the inverse of the summed *p*-values of the two barriers it connects, with edges below a strength of 2 excluded. All statistical analyses were performed using R (version 4.4.2, Vienna, Austria), and a *p*-value of <0.05 was considered statistically significant.

## 3. Results

### 3.1. Patient Perspective

Three hundred and fifteen patients with CD completed the questionnaire. The mean age was 36.7 (SD ± 16). Descriptive characteristics are presented in Table S1. Seventy-two percent reported being treated/initiating treatment with advanced therapy (biologic or small-molecule therapy). Moreover, 72% previously required CD-related hospitalization, while 32.7% required CD-related surgery. At the time of the questionnaire, 42% of the patients were in clinical remission, 36% had active disease, and 22% were not sure of their disease status.

Previous EEN exposure was reported by 38.7% (*n* = 122/315) of the patients, with varying treatment duration, while 58.7% of these patients (*n* = 71/122) perceived a beneficial effect from EEN. Sixty-one percent (*n* = 192/315) had heard about EEN as a therapeutic method. However, the sources of information were diverse, and 53.7% (*n* = 169/315) were interested in learning more, preferentially from their physician (87.6%, *n* = 148/169), as well as from other staff members [a dietitian (43.8%, *n* = 74/169) and an IBD nurse (26.6%, *n* = 45/169)]. Reluctance to try EEN was reported by 20.3% (i.e., they replied ‘No’, while 48% replied ‘Yes’ and 31.7% replied ‘Maybe’). In univariate and multivariate analysis, current advanced therapy was associated with EEN reluctance (OR 2.06 [95%CI 1.05–4.35], Tables S2 and S3).

### 3.2. Barriers and Support Factors

**Figure 1:**
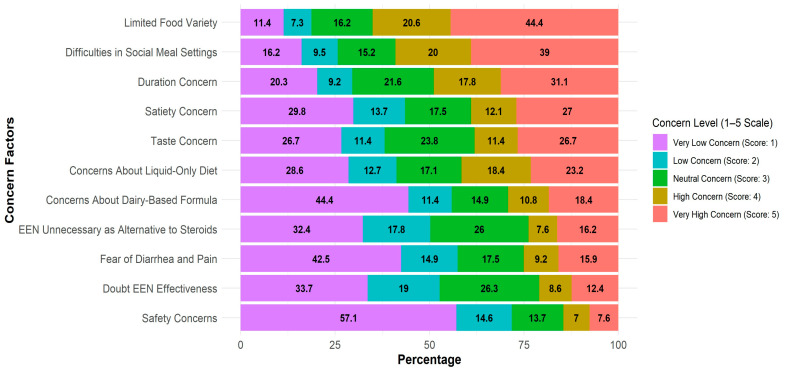
Barriers affecting patients’ reluctance to try EEN. Likert score distribution (1—very low to 5—very high concern) of concern factors among patients. Each bar represents 100% of patients (*n* = 315), the color represents the score, and the numbers in the graph represent the % of patients who reported the score.

Top patient-ranked barriers to EEN were ‘lack of diversity’ (65% scored 4–5), ‘difficulty of participating in family and social meals’ (59% of respondents scored 4–5), and treatment duration (49% of respondents scored 4–5). The respondents were less concerned about potential complications (14.6% of respondents scored 4–5), treatment-related diarrhea and pain (25.1% of respondents scored 4–5), and the notion that the formula is dairy-sourced (29.2% of respondents scored 4–5) (Figure 1).

**Figure 2:**
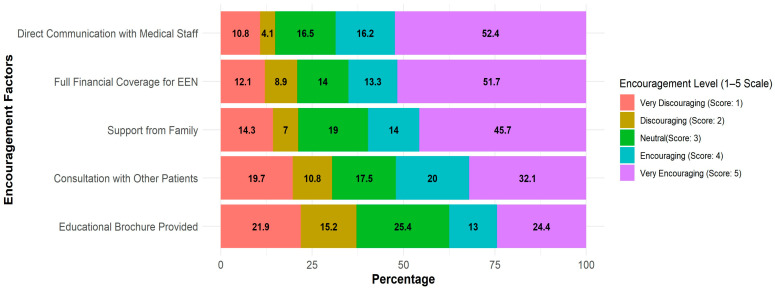
Supportive factors affecting patients’ reluctance to try EEN. Likert score distribution of support factors among patients (1—very discouraging to 5—very encouraging). Each bar represents 100% of patients (*n* = 315), the color represents the score, and the numbers in the graph represent the % of patients who reported the score.

Supportive factors that could facilitate EEN use were as follows: direct communication with a staff member (68.6% of respondents scored 4–5), full funding of the formula (65% of respondents scored 4–5), family support (59.7% of respondents scored 4–5), and talking with other patients that are EEN-experienced (52% of respondents scored 4–5) (Figure 2).

### 3.3. Physician Perspective

Forty-two gastroenterologists completed the questionnaire. Baseline characteristics and past EEN experience are presented in Table S4. Seventy-one percent (*n* = 30/42) of the physicians have recommended EEN as a treatment option. However, only 65% of these physicians (*n* = 19/30) have done so several times. Indications for EEN therapy were diverse and included complicated disease, malnutrition, and mild-to-moderate disease. Seventy-six percent (*n* = 32/42) of physicians had encountered patients with EEN experience, and only nine percent reported it as being not beneficial. Nevertheless, when we assessed the physicians’ knowledge of EEN-achieved remission rates in adult patients, responses varied widely: 40% (*n* = 17/42) of respondents believed that the remission rate ranged between 30 and 60%, 33% (*n* = 14/42) assessed it as 0–30%, and 26% (*n* = 11/42) assessed it as 60–80%.

Eighty-two percent (*n* = 34/42) of the physicians expressed willingness to prescribe EEN for their patients. In both univariate and multivariate analyses, previous EEN prescription was associated with willingness to prescribe EEN (OR 4.67, CI 95% 1.11–19.54), while the frequency of addressing the nutritional aspect during clinic visits was associated with higher willingness to prescribe EEN in univariate analysis (*p* = 0.013) but not in multivariate analysis (Tables S5 and S6). Twenty-six percent of respondents (26.2%, *n* = 11/42) reported that they felt uncomfortable offering this treatment method.

**Figure 3:**
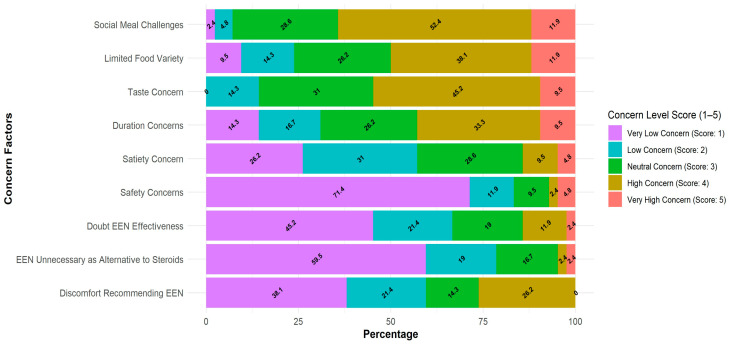
Barriers affecting physicians’ willingness to prescribe EEN and their perceived patients’ barriers. Likert score distribution (1—very low to 5—very high concern) of concern factors among patients. Each bar represents 100% of physicians (*n* = 42), the color represents the score, and the numbers in the graph represent the % of physicians who reported the score.

When examining the perceived patient-related barriers among physicians, the social factor was perceived as the most significant obstacle (64.3% of respondents scored 4–5), followed by the formula taste (54.7% scored 4–5), lack of food diversity (50% scored 4–5), and treatment duration (42.8% scored 4–5). The majority of respondents were not concerned about safety (7.2% scored 4–5) or about the efficacy of the treatment (14.3% scored 4–5) (Figure 3).

### 3.4. Physician–Patient Gaps in EEN Treatment

**Figure 4:**
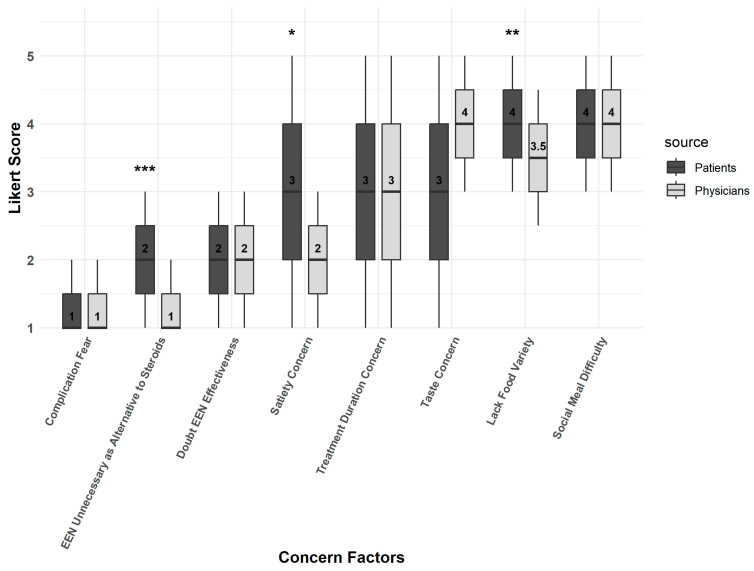
Perceived barriers among patients and physicians. Box plot showing the Likert scores for barriers to EEN, comparing physicians and patients. The central line represents the median, box margins indicate the IQR, and whiskers extend across the full range of Likert scores (1–5). Differences between physicians and patients were assessed using the Mann–Whitney U test. Statistical significance is indicated as *—*p* < 0.05, **—*p* < 0.01, and ***—*p* < 0.001.

Overall, the physicians’ perspectives largely aligned with those of the patients. However, we found that ‘lack of food variety’ and ‘hunger concerns’ were barriers which were scored significantly higher by patients than by physicians (Figure 4).

**Figure 5:**
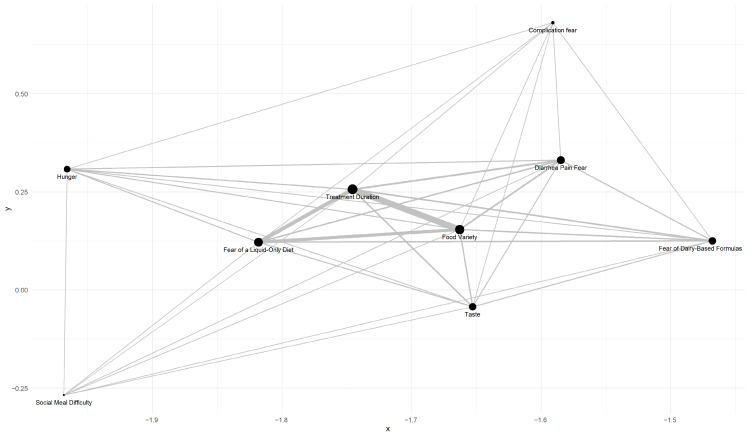
Relationship between patients’ barriers. Kendall’s Tau Correlation Network represents the relationships between barriers and EEN reluctance (nodes), as well as the correlations between barriers (edges). Node size represents Kendall’s Tau correlation coefficient scaled by 100, while edge strength is defined as the inverse of the summed *p*-values of the two barriers it connects, with edges below a strength of 2 excluded. Line width denotes edge strength (thicker = stronger); strength is the inverse of the sum of the two p-values, and edges with strength < 2 are omitted.

Since barriers can be expected to interact with each other in an uncontrolled environment, we used a network plot for visualization. As shown in Figure 5, the top three barriers associated with EEN reluctance were ‘food variety’, ‘treatment duration’, and ‘fear of having a liquid-only diet’, which correlated with each other.

## 4. Discussion

In our study, 80% of participants expressed openness to discuss EEN therapy while 48% were willing to try it. Among the barriers evaluated, lack of food diversity, difficulties participating in social and family meals, and the long duration of EEN treatment were perceived by patients as the most significant hurdles to EEN use. Conversely, direct communication with a staff member, funding of EEN therapy, and strong family support were identified as key factors that could encourage patients to pursue EEN. From the physicians’ perspective, 82% of gastroenterologists indicated that they would be willing to prescribe EEN, and this was strongly associated with prior experience prescribing it. In practice, most reported offering EEN only infrequently, and only a minority had considered it for patients with penetrating disease. Given EEN’s demonstrated safety and efficacy, these results suggest that it should be more consistently offered to patients.

Exclusive enteral nutrition is highly effective in inducing remission in pediatric patients with CD and is considered a first-line therapy [15]. Although lower response rates have been reported in adult patients, it is still considered an effective therapy, with variability across studies and geographic locations [16]. In fact, the most recent CD therapy consensus by ECCO includes EEN in the therapeutic tools to induce remission in select CD patients [8]. Importantly, studies show that when adherence is high, the response rates are similar to those seen in the pediatric population [16]. Therefore, improving adherence and proper patient selection are crucial elements in expanding the use of EEN in adult care. Similarly to our findings, a previous review indicated that a common reason for withdrawal was low palatability of the nutritional therapy [10].

Our survey specifically highlights patients’ concerns with the social effect of EEN, poor food palatability, and low food diversity. These findings echo a qualitative interview-based study, in which participants described the first days as the most challenging, with many missing the rewarding experience of having solid food by the end of the eight-week regimen [17]. The social factor emerged as the most challenging aspect, aligning with our findings. In the study, patients coped with this challenge in different ways: some avoided social meals entirely, others brought their formula to social gatherings, and some initially avoided joint meals but later participated once they adjusted to the diet. Strong family and social support and continuous feedback and communication with treating staff were mentioned as facilitators of adherence in that cohort as well [17], highlighting the need for structured social adaptation strategies and strong multidisciplinary support. Similarly, in a national Spanish survey of 51 medical centers, lack of time, lack of social support, and inadequate follow-up by a multidisciplinary staff were reported as the most significant barriers to EEN adherence [13]. In addition to these psychosocial and structural challenges, economic considerations play a crucial role. Financial coverage of EEN costs has been identified as a key factor in enhancing adherence, and should therefore be addressed at the national policy level. Similarly, time commitment—especially in the context of prolonged dietary restrictions—poses a significant challenge for many patients. Accordingly, shortening the duration of EEN is another strategy to improve adherence. Studies have shown that a shorter EEN course (2 weeks vs. 6 weeks) significantly improves compliance rates (85% vs. 67%, respectively) [16]. This is based on pediatric studies that demonstrated significant clinical and biochemical remission rates after only 2–3 weeks of EEN [9,18]. While some studies in adult patients reported 80% remission rates after 4–6 weeks [16], similar remission rates were achieved within as little as 2 weeks [19]. The individual variation in time to response suggests that personalized treatment duration may enhance patient compliance without compromising efficacy. However, more research is needed to support this option. Improving enteral nutrition palatability and diversity is another important strategy to support adherence. Novel nutritional interventions such as the CD Elimination Diet (CDED) can address patients’ concerns [20].

Among the past therapy exposure variables examined, only current use of advanced therapy was significantly associated with EEN reluctance. This may reflect patients’ perception that nutritional therapy is more ‘natural’, especially among patients inclined to avoid pharmacological treatments. However, emerging evidence suggests that a combination of EEN with biological therapy can improve outcomes in adult patients, and therefore should be offered to this patient population as well [21]. Interestingly, we found that previous EEN exposure or past positive experience with EEN was not associated with EEN acceptability or EEN reluctance. This appears to be in contrast to a previous study where past parenteral exposure was highly associated with patients’ acceptance of oral, intravenous, and subcutaneous therapy [22]. These differences may point to unique perceptions surrounding nutritional interventions or highlight gaps in education and counseling regarding EEN as a viable adjunctive therapy in adult care.

The results of the physician survey highlight the underutilization of EEN among physicians, despite the overall positive attitude toward it. A possible explanation for the gap between low EEN use and positive attitudes toward it is that nutritional therapy is an orphan intervention, not advocated by the pharmaceutical industry, thus explaining the variability in perceived EEN efficacy among physicians. We believe that to increase physicians’ acceptability of EEN, more nutritional research on EEN efficacy in IBD and increasing visibility of the results in scientific meetings and guidelines would be beneficial. Indications for EEN use should be broadened to more complicated cases as well, since EEN can also promote enterocutaneous fistula closure [11,23,24] and healing of inflammatory stricture [23,24], prevent surgery [9,23,24,25], and reduce postoperative complications [26].

We were encouraged to find that patients’ and physicians’ perspectives were largely aligned, although significant differences regarding hunger sensation and lack of food variety were noted. The positive attitude toward EEN shared by patients and physicians can be explained by the high percentage of physicians with good familiarity and experience in prescribing EEN therapy and the high proportion of physicians working in an academic hospital setting with IBD centers. Our study has several limitations. First, our findings on physicians’ attitudes toward EEN may not represent attitudes of community gastroenterologists. Additional limitations include selection bias due to the use of digital forms, which may have favored younger, more digitally literate respondents, although efforts were made to assist older, less digitally literate participants in completing the questionnaire. Another limitation is the potential for recall bias, which may have influenced the first section of the questionnaire, although the impact of recall bias on this study’s major aims should be minimal. Another limitation was that patient characteristics and the clinical setting of participating centers suggest that our study population may represent a subgroup with more severe disease compared to the general CD population and thus that our findings may be more applicable to referral centers. Lastly, as this was an observational survey study, our analyses were exploratory in nature and should be interpreted with caution. While we aimed to comprehensively characterize factors that might influence EEN utilization, the modest sample size of the physician group (*n* = 42) and the relatively small subgroup of reluctant patients may have limited statistical power, increasing the risk of type II errors (false negatives). Conversely, the number of comparisons performed also raises the possibility of type I errors (false positives), which should be considered when interpreting individual findings. Although we employed both univariate and multivariate approaches, the latter included a limited set of available clinical and demographic covariates, and residual confounding cannot be excluded. Given these constraints, our findings should be regarded as hypothesis-generating rather than confirmatory. Nevertheless, we believe that the results reflect real-world challenges that both patients and physicians face regarding EEN and, given the relatively large overall cohort compared to prior studies, provide important insights into barriers and opportunities for improving its use in clinical practice.

## 5. Conclusions

In summary, our findings underscore the need to offer EEN more frequently as a viable therapeutic option in adult CD management while addressing patient concerns regarding EEN palatability, cost, and social effects. Enhanced patient education, stronger multidisciplinary support, and potential implementation of shorter EEN courses may improve compliance. Given the evidence supporting the efficacy of EEN in the adult CD population, future prospective studies are needed to explore optimal implementation strategies and assess its role alongside advanced therapies.

## Data Availability

The data provided by the authors is not part of a repository or public database. The PI (RMR) will be happy to discuss sharing the data with interested researchers upon request.

## Supplementary Materials

The following supporting information can be downloaded at https://www.mdpi.com/article/10.3390/nu17182945/s1. File S1: Patient Questionnaire (translated to English); File S2: Physician Questionnaire; Table S1: Patients characteristics; Table S2: Patients’ Characteristics Stratified by Reluctance to Try EEN; Table S3: Multivariate Analysis of Patients’ EEN Reluctance; Table S4: Physicians’ Baseline Characteristics and EEN Experience; Table S5: Physicians’ Characteristics Stratified by Willingness to Prescribe EEN; Table S6: Physicians’ Characteristics Stratified by Willingness to Prescribe EEN.

## Abbreviations

EEN	Exclusive enteral nutrition
CD	Crohn’s disease
OR	Odds ratio
SD	Standard deviation
CDED	Crohn’s disease elimination diet
