# Supplementary material for: Understanding Patient and Physician Perspectives on Exclusive Enteral Nutrition in Adults with Crohn’s Disease: Bridging the Gap in Nutritional Therapy
Source: Nutrients. 2025 Sep 12;17(18):2945. doi: 10.3390/nu17182945 (PMC12473139; doi:10.3390/nu17182945)
Supplement: Supplementary file 1 [file nutrients-17-02945-s001.zip › File S1.pdf]

File S1: Patient Questionnaire (translated to English)

Hello,

Before you is a short questionnaire which is part of a study aimed at better understanding IBD patient attitudes to the Exclusive Enteral Nutrition (EEN) treatment regimen. The EEN formula (Ensure/Modulen) is a treatment regimen lasting ~6-8 weeks during which the patient consumes exclusively this formula, and it is forbidden to consume any other food during this period. Studies have demonstrated that this diet is effective in controlling the inflammatory process and can be used instead of corticosteroids until beginning an established treatment regimen such as with biologic or immunomodulatory drugs.

Our goal in this research is to discover the extent to which patients would be willing to try this approach instead of corticosteroids for the purpose of lowering inflammation.

We would be much obliged if you could take around 3 minutes to fill in the following questionnaire. Answering the questionnaire constitutes your consent to participate in this study. Participation in the survey is optional and it is your choice to fill it out or not. It is important to note that there is no direct impact from participating in the survey and answering the questionnaire. The questionnaire is completely anonymous and will be used exclusively for the aforementioned research purposes.

Thank you very much for your cooperation.

A. General Details

1. Age:*

   Your answer

2. Sex:*

   a) Male

b) Female

c) Other

3. Ethnic Group:*

a) Muslim

b) Christian

c) Jewish

d) Druze

e) Other

4. Marital Status:*

a) Single

b) Married

c) Divorced

5. Education:*

a) Less than 12-years

b) 12 years

c) Academic

6. At what age were you diagnosed with Crohn's disease?*

Your answer

7. Were you ever hospitalized due to intestinal illness?*

a) Yes

b) No

8. Did you ever need surgery due to intestinal illness?*

a) Yes

b) No

9. What treatment are you receiving today? (Check all that apply)*

- Nutritional therapy only
- Immunomodulatory agents (methotrexate, imuran, puri-nethol)
- Biologic treatment (humira, remicaid, stelara, entyvio, etc.)
- Not receiving treatment
- Other:

10. Is the illness currently active or in remission?*

a) Remission

b) Active

c) Unsure

B. Exclusive Enteral Nutrition with Formula (Ensure/Modulen)

11. Have you ever heard of EEN as a treatment for Crohn's disease?*

a) Yes

b) No

12. If you answered "yes" to the previous question, from whom did you hear about it?

(Check all that apply)

- A friend or relative
- Physician
- IBD nurse
- Dietitian
- Online (Google, etc.)
- Social media
- Other:

13. Would you like to hear more information about this treatment option?*

a) Yes

b) No

14. If you answered "yes" to the previous question, from whom would you like to receive this information?

a) Nurse

b) Physician

c) Dietician

d) Other:

15. Have you ever tried this treatment in the past?*

a) Yes

b) No

16. If you answered "yes" to the previous question, for what duration did you receive this treatment?

a) 2 weeks

b) 4 weeks

c) 6 weeks

d) 8 weeks

17. If you have previously tried this type of treatment in the past, did you have a good response to it?

a) Yes

b) No

c) Maybe

18. Were you able to keep up with the treatment plan through the end?

a) Yes

b) No

19. If you answered "no" to the previous question, what was the reason?

Your answer

20. The EEN formula (Ensure/Modulen) treatment protocol lasts for the duration of **6-8 weeks** during which the patient is nourished **exclusively** with the formula, and it is forbidden **to consume any other food items during this period.**

Studies have shown that this diet is effective in attaining control over the inflammatory process and it can be used **in lieu of corticosteroids** until beginning a set medical treatment plan, such as biologic medications or immunomodulatory agents.

If this treatment protocol was suggested to you **instead of corticosteroids** in order to stop the inflammation, would you be willing to try it?*

a) Yes

b) No

c) Maybe

21. What would stop you from trying this treatment? (please rank the following reasons from 1 to 5, with 1 being the least, and 5 being the most significant):

The concern that it would not be appetizing:*

1 2 3 4 5

The lack of variety in the diet - it would be too difficult to always eat the same thing:*

1 2 3 4 5

The treatment time is too long, and I am unsure I would be able to withstand it:*

1 2 3 4 5

I am concerned that I would be hungry:*

1 2 3 4 5

I do not believe it would help me:*

1 2 3 4 5

I am concerned that this treatment approach may have health risks for me:*

1 2 3 4 5

I am concerned that this treatment might make my diarrhea and stomach pains worse:*

1 2 3 4 5

The difficulty with attending family and social meals, including at work:*

1 2 3 4 5

I do not feel that I would need this:*

1 2 3 4 5

I am concerned that this would be a liquid diet without the option to eat solid food:*

1 2 3 4 5

I am concerned about the implications of the formula having dairy ingredients:*

1 2 3 4 5

22. In the event that this treatment was suggested to you for a duration less than 6 weeks, would you choose it over corticosteroids?*

a) Yes
b) No
c) Maybe

23. If you answered "yes" to the previous question, what would be the timeframe you think would be reasonable for this treatment?*

a) 2 weeks
b) 4 weeks
c) 5 weeks

24. Please rank the following options on a scale of 1-5, with 1 being "would not help me", and 5 being "would help me very much":

Family support:*

1 2 3 4 5

Information booklets on the topic:*

1 2 3 4 5

Complete coverage of the expense of the formula (i.e. free of charge):*

1 2 3 4 5

Talking to other patients who have tried this treatment in the past:*

1 2 3 4 5

Direct contact with a staff member (dietician, nurse, doctor, etc.):*

1 2 3 4 5
