# Supplementary material for: Understanding Patient and Physician Perspectives on Exclusive Enteral Nutrition in Adults with Crohn’s Disease: Bridging the Gap in Nutritional Therapy
Source: Nutrients. 2025 Sep 12;17(18):2945. doi: 10.3390/nu17182945 (PMC12473139; doi:10.3390/nu17182945)
Supplement: Supplementary file 1 [file nutrients-17-02945-s001.zip › File S2.pdf]

File S2: Physician Questionnaire

Greetings,

Before you is a short questionnaire, which is part of an investigation into the opinions of physicians and their adult IBD patients on the use of exclusive enteral nutrition.

We would greatly appreciate it if you could dedicate approximately 3 minutes of your time to fill out our questionnaire.

Your completion of the questionnaire constitutes your agreement to participate in our investigation. Nevertheless, participation in the survey is entirely voluntary and you may choose to participate or not. It is important to note that there is no direct impact from participating in the survey and answering the questionnaire. The questionnaire is completely anonymous and will be used exclusively for the aforementioned research purposes.

Thank you very much for your cooperation,

Dr. Ramit Magen-Rimon: r_magen@rmc.gov.il

Dr. Haggai Bar-Yosef: h_bar-yoseph@rmc.gov.il

A. General Details

1. Age:*

   Your answer

2. Gender:*

   a) Male

   b) Female

   c) Other

3. Ethnic group:*

   a) Muslim

   b) Christian

   c) Jewish

d) Druze

e) Other

4. What year did you complete your specialization in gastroenterology?*

Your answer

5. In what medical center are you employed?*

a) HaEmek

b) Poriya

c) Rebecca Ziv

d) Nahariya Medical Center

e) Bnei Zion-Rothschild

f) Carmel

g) Rambam

h) Hillel Yaffe

i) Meir

j) Shaarei Tzedek

k) Hadassah

l) Wolfson

m) Sheba

n) Ichilov

o) Shamir Medical Center

p) Beilinson

q) Kaplan

r) Assuta Ashdod

s) Soroka

t) Health Maintenance Organizations

6. How many years have you been treating IBD patients?*

a) <5

b) 5-10

c) 10-15

d) 15-20

e) >20

7. How many IBD patients do you treat per year?*

a) <10

b) 10-30

c) 31-60

d) 61-100

e) >100

8. Where did you study medicine?*

a) Outside Israel

b) Israeli faculties

9. If you completed a fellowship outside Israel, where did you do it?

a) United States

b) Canada

c) Europe

d) Australia

e) Other:

10. Did you complete a rotation in pediatric gastroenterology as part of your residency?*

a) Yes

b) No

11. Do you regularly participate in pediatric gastroenterology research, meetings, or other procedures?*

a) Yes

b) No

B. Exclusive Enteral Nutrition - (Modulen/Ensure)

12. Have you ever recommended EEN to your patients as part of their treatment for Crohn's disease?*

a) Yes

b) No

13. If you answered "yes" to the previous question, approximately how many times have you recommended the use of EEN in the past?

Your answer

14. Do you have patients who have tried this treatment in the past?*

a) Yes

b) No

c) I am unsure

15. If you answered "yes" to the previous question, was there a good response to the treatment?

a) Yes

b) No

c) In some cases, yes and in some no

If you were to recommend this treatment to patients of yours, for which patients would you recommend it? (Check all that apply)

- Patients with mild to intermediate disease
- Patients with intermediate to severe disease
- Malnourished patients
- Patients with invasive disease
- Patients with restricted disease

17. With what frequency do you address nutritional issues with your patients*

a) At every visit

b) At the majority of visits

c) At some visits

d) Only if the patient brings up the issue

e) Never

18. From your knowledge of the literature, what is the percentage of adults in whom EEN has successfully induced remission? *

a) 0-30

b) 30-60

c) 60-80

d) 80-100

19. What, in your eyes, are the major obstacles to giving EEN? (Rank the reasons from 1-5, with 1 being insignificant and 5 being most significant)

I don't feel comfortable prescribing this diet:*

1 2 3 4 5

I think patients will refuse this diet due to its unpalatability:*

1 2 3 4 5

I think patients will refuse this diet due to the lack of variety:*

1 2 3 4 5

I think patients will refuse this diet due to the difficulties it poses on family and social meals, including at work:*

1 2 3 4 5

The treatment time is too long (6-8 weeks):*

1 2 3 4 5

I think it is impossible to feel satisfied on this diet:*

1 2 3 4 5

I do not believe that it will help the patient:*

1 2 3 4 5

I worry that the treatment may harm the patient:*

1 2 3 4 5

I think that the treatment is unnecessary given the existence of other options like corticosteroids:*

1 2 3 4 5

20. In the event that this treatment protocol was to be offered for a shorter duration than 6 weeks, would you choose it instead of corticosteroids?*

a) Yes

b) No

c) Maybe

21. If you answered "yes" to the previous question, what would be the duration you think would be reasonable to try this treatment for?

Your answer

22. I feel that I do not have enough knowledge or experience in order to recommend such a diet:*

1 2 3 4 5

23. Are there professionals from the dietary discipline available to you which you could turn to for help guiding and accompanying the patients? (dieticians or doctors who specialize in the field)*

a) Yes

b) No

24. If there was support and accompaniment available to you from the dietary perspective, would you recommend EEN?*

a) Yes

b) No

c) Maybe

25. Do you recommend other dietary approaches in the treatment of your patients, such as CDED, LOW FODMAP, or others?*

a) Yes

b) No
