# Supplementary material for: Understanding Patient and Physician Perspectives on Exclusive Enteral Nutrition in Adults with Crohn’s Disease: Bridging the Gap in Nutritional Therapy
Source: Nutrients. 2025 Sep 12;17(18):2945. doi: 10.3390/nu17182945 (PMC12473139; doi:10.3390/nu17182945)
Supplement: Supplementary file 1 [file nutrients-17-02945-s001.zip › Table S1.pdf]

Table S1 – Patients characteristics

	Overall (N=315)
**Age,** Years, Mean (SD)	36.7 (16.0)
**Female**, n (%)	180 (57.1)
**Ethnicity,** n (%)	
Jewish	259 (82.7)
Arab	42 (13.4)
Druze	9 (2.9)
Other	3 (1.0)
**Marital Status,** n (%)	
Single	166 (53)
Married	130 (41.5)
Divorced	19 (6.0)
**Education,** n (%)	
Less than 12 years	12 (3.8)
High School Graduate	122 (38.7)
More than 12 years	181 (57.5)
**Age Diagnosed with CD**, Years, Mean (SD)	24.7 (14.2)
**Disease Duration,** Years**,** Median [Range]	10 [0, 60]
**Past Hospitalization for CD,** n (%)	224 (71.1)
**History of CD-Related Surgery,** n (%)	103 (32.7)
**Currently Receiving CD Treatment,** n (%)*	249 (79.0)
**Corticosteroids,** n (%)	7 (2.2)
**Immunomodulators,** n (%)	28 (8.9)
**Advanced therapy,** n (%)	227 (72.1)
**Previously Heard of EEN,** n (%)*	193 (61.3)
**Heard of EEN from a Friend,** n (%)	31 (16.1)
**Heard of EEN on Google,** n (%)	33 (17.1)
**Heard of EEN from Social Media,** n (%)	34 (17.6)
**Heard of EEN from a Doctor,** n (%)	116 (60.1)
**Heard of EEN from an IBD Nurse,** n (%)	36 (18.7)
**Heard of EEN from a Dietitian,** n (%)	71 (36.8)
**Wish to Hear More about EEN,** n (%)*	169 (53.7)
**Prefer to Hear More from a Doctor,** n (%)	148 (87.6)
**Prefer to Hear More from a Nurse,** n (%)	45 (26.6)
**Prefer to Hear More from a Dietitian,** n (%)	74 (43.8)
**Past EEN Exposure,** n (%)	122 (38.7)
**Past EEN Duration,** n (%)**	
2 Weeks	23 (18.1)
4 Weeks	12 (9.4)
6 Weeks	23 (18.1)
>=8 Weeks	69 (54.3)
**Perceived Clinical Response to EEN,** n (%)**	
Yes	71 (58.7)
No	24 (19.8)
Maybe	26 (21.5)
**Previous EEN Regimen Completion,** n (%)	77 (64.2)

Values represent n (%); SD - standard deviation; EEN - exclusive enteral nutrition, CD - Crohn's disease; IBD - inflammatory bowel disease.
*Percentages reflect the total number of responses rather than individual respondents, as multiple selections were allowed.
**Not all patients who answered affirmatively to previous EEN exposure may have answered the following questions.
