# Supplementary material for: Understanding Patient and Physician Perspectives on Exclusive Enteral Nutrition in Adults with Crohn’s Disease: Bridging the Gap in Nutritional Therapy
Source: Nutrients. 2025 Sep 12;17(18):2945. doi: 10.3390/nu17182945 (PMC12473139; doi:10.3390/nu17182945)
Supplement: Supplementary file 1 [file nutrients-17-02945-s001.zip › Table S2.pdf]

Table S2 - Patients' Characteristics Stratified by Reluctance to Try EEN

	EEN Receptive (N=251)	EEN Reluctant (N=64)	P-value
**Age,** Years, Mean (SD)	36.6 (16.0)	37.0 (18.0)	0.871
**Female**, n (%)	140 (55.8)	40 (62.5)	0.407
**Ethnicity,** Jewish, n (%)	211 (84.1)	48 (75.0)	0.131
**Married**, n (%)	102 (40.6)	28 (43.8)	0.757
**Education, 12+ years**, n (%)	241 (96.0)	62 (96.9)	1
**Age at CD Diagnosis,** Years, Mean (SD)	24.3 (13.5)	26.6 (16.7)	0.251
**Disease Duration,** Years, Median [Range]	10.0 [0, 60.0]	8.00 [0, 36.0]	0.186
**Previous Hospitalization for CD,** n (%)	180 (71.7)	44 (68.8)	0.755
**History of CD-Related Surgery,** n (%)	86 (34.3)	17 (26.6)	0.306
**Active Disease**, n (%)	83 (33.1)	28 (43.8)	0.186
**Current Treatment,** n (%)	194 (77.3)	55 (85.9)	0.179
**Corticosteroids,** n (%)	6 (2.4)	1 (1.6)	1
**Immunomodulators,** n (%)	24 (9.6)	4 (6.3)	0.559
**Advanced therapy,** n (%)	174 (69.3)	53 (82.8)	**0.046**
**Past Experience with EEN,** n (%)	101 (40.2)	21 (32.8)	0.345

Values represent n (%); SD - standard deviation; EEN - exclusive enteral nutrition; CD - Crohn's disease. Advanced therapy represents biological or small molecule therapy.
