# Supplementary material for: Understanding Patient and Physician Perspectives on Exclusive Enteral Nutrition in Adults with Crohn’s Disease: Bridging the Gap in Nutritional Therapy
Source: Nutrients. 2025 Sep 12;17(18):2945. doi: 10.3390/nu17182945 (PMC12473139; doi:10.3390/nu17182945)
Supplement: Supplementary file 1 [file nutrients-17-02945-s001.zip › Table S3.pdf]

Table S3 - Multivariate Analysis of Patients' EEN Reluctance

	Coefficient	Odds Ratio	95% CI	P-value
**Age**	0.001	1.001	0.985 - 1.018	0.870
**Female**	0.279	1.321	0.752 - 2.323	0.333
**Jewish ethnicity**	-0.564	0.569	0.294 - 1.099	0.093
**Marital status, Single**	-0.128	0.880	0.506 - 1.532	0.652
**> 12 years of education**	0.252	1.286	0.275 - 6.022	0.749
**Age at CD Diagnosis**	0.008	1.008	0.989 - 1.028	0.388
**Previous Hospitalization for CD**	-0.142	0.868	0.478 - 1.574	0.641
**History of CD-Related Surgery**	-0.365	0.694	0.376 - 1.281	0.243
**Active CD**	0.454	1.57	0.900 - 2.755	0.110
**Past Experience with EEN**	0.321	1.379	0.772 - 2.461	0.277
**Perceived Positive Response to EEN**	0.610	1.841	0.887 - 3.823	0.101
**Currently Receiving Corticosteroids**	-0.434	0.648	0.077 - 5.482	0.691
**Currently Receiving Immunomodulator Therapy**	-0.461	0.631	0.211 - 1.887	0.410
**Currently Receiving Advanced Therapy**	0.757	2.132	1.056 - 4.305	**0.035**

CI - confidence interval, EEN - exclusive enteral nutrition, CD Crohn's disease; 5-ASA – 5 aminosalicylate. Advanced therapy represents biological or small molecule therapy.
