# Supplementary material for: Understanding Patient and Physician Perspectives on Exclusive Enteral Nutrition in Adults with Crohn’s Disease: Bridging the Gap in Nutritional Therapy
Source: Nutrients. 2025 Sep 12;17(18):2945. doi: 10.3390/nu17182945 (PMC12473139; doi:10.3390/nu17182945)
Supplement: Supplementary file 1 [file nutrients-17-02945-s001.zip › Table S4.pdf]

Table S4 - Physicians' Baseline Characteristics and EEN Experience

	Overall (N=42)
**Age,** Years, Mean (SD)	48.7 (8.0)
**Female**, n (%)	22 (52.4)
**Ethnicity**, Jewish, n (%)	37 (88.1)
**Years in GI Practice,** Median [Range]	5 [5, 35]
**Medical practice setting**, n (%)	
Hospitals	39 (92.9)
Community Clinics	3 (7.1)
**Years Treating IBD,** n (%)	
<5	9 (21.4)
5-10	12 (28.6)
10-15	8 (19.0)
15-20	7 (16.7)
>20	6 (14.3)
**Estimated IBD Patients Per Year,** n (%)	
<10	2 (4.8)
10-30	12 (28.6)
31-60	11 (26.2)
61-100	6 (14.3)
>100	11 (26.2)
**Medical School Attended,** n (%)	
Israeli Faculties	34 (81.0)
Foreign Faculties	8 (19.0)
**International Fellowship,** n (%)	21 (50.0)
**Location of International Fellowship,** n (%)	
Australia	3 (7.1)
Europe	5 (11.9)
USA	12 (28.6)
Canada	1 (2.4)
**Pediatric Gastroenterology Rotation,** n (%)	
Yes	1 (2.4)
No	41 (97.6)
**Pediatric Gastroenterology Collaboration,** n (%)	
Yes	24 (57.1)
No	18 (42.9)
**Previous EEN Recommendation,** n (%)	
Yes	30 (71.4)
No	12 (28.6)
**Instances of Previous EEN Employment,** n (%)	
<5	15 (35.7)
5-10	5 (11.9)
10-15	2(4.8)
>15	11 (26.2)
**Encounters with EEN Experienced Patients,** n (%)	
Yes	32 (76.2)
No	7 (16.7)
Unsure	3 (7.1)
**Patients with Positive Past EEN Experience,** n (%)	
Yes	15 (45.5)
No	3 (9.1)
Mixed	15 (45.5)
**Indication for EEN Recommendation,** n (%)*	
Mild to moderate disease	14 (41.2)
Moderate to severe disease	19 (55.9)
Patients with malnutrition	19 (55.9)
Patients with penetrating disease	13 (38.2)

Patients with stricturing disease	18 (52.9)
**Frequency Addressing Nutrition,** n (%)	
On every visit	15 (35.7)
On most visits	18 (42.9)
On some visits	8 (19.0)
Only per patient request	1 (2.4)
Never	0 (0.0)
**Physician's Estimate of EEN Remission Rates,** n (%)	
0-30%	14 (33.3)
30-60%	17 (40.5)
60-80%	11 (26.2)
80-100%	0 (0.0)

Values represent n (%); SD - standard deviation; EEN - exclusive enteral nutrition; IBD - inflammatory bowel disease.
*Percentages reflect the total number of responses rather than individual respondents, as multiple selections were allowed.
