# Supplementary material for: Understanding Patient and Physician Perspectives on Exclusive Enteral Nutrition in Adults with Crohn’s Disease: Bridging the Gap in Nutritional Therapy
Source: Nutrients. 2025 Sep 12;17(18):2945. doi: 10.3390/nu17182945 (PMC12473139; doi:10.3390/nu17182945)
Supplement: Supplementary file 1 [file nutrients-17-02945-s001.zip › Table S5.pdf]

Table S5 - Physicians' Characteristics Stratified by Willingness to Prescribe EEN

	Recommend EEN (N=19)	Do Not Recommend EEN (N=23)	P-value
**Age,** Years, Mean (SD)	49.2 (7.37)	48.3 (8.67)	0.727
**Female**, n (%)	19 (63.3%)	3 (25.0%)	0.0568
**Jewish**, n (%)	17 (89.5%)	20 (87.0%)	1
**Years in GI Practice**, Median [Min, Max]	2020 [1990, 2020]	2020 [2000, 2020]	0.702
**Medical Center Affiliation,** n (%)			1
Hospitals	17 (89.5%)	21 (91.3%)	
Community Clinics	2 (10.5%)	2 (8.7%)	
**Years Treating IBD,** n (%)			0.756
<5	5 (26.3%)	4 (17.4%)	
5-10	4 (21.1%)	8 (34.8%)	
10-15	4 (21.1%)	4 (17.4%)	
15-20	4 (21.1%)	3 (13.0%)	
>20	2 (10.5%)	4 (17.4%)	
**IBD Patients Per Year,** n (%)			0.937
<10	1 (5.3%)	1 (4.3%)	
31-60	5 (26.3%)	6 (26.1%)	
61-100	2 (10.5%)	4 (17.4%)	
>100	6 (31.6%)	5 (21.7%)	
**Medical School Attended,** n (%)			0.925
Israeli Schools	16 (84.2%)	18 (78.3%)	
Foreign Schools	3 (15.8%)	5 (21.7%)	
**Location of International Fellowship,** n (%)			0.477
United States	4 (21.1%)	8 (34.8%)	
Europe	3 (15.8%)	2 (8.7%)	
Canada	1 (5.3%)	0 (0%)	
Australia	1 (5.3%)	2 (8.7%)	
**Past Pediatric Gastroenterology Rotation,** n (%)	0 (0%)	1 (4.3%)	1
**Pediatric Gastroenterology in Collaboration,** n (%)	9 (47.4%)	15 (65.2%)	0.395
**Previous EEN Recommendation,** n (%)	15 (78.9%)	15 (65.2%)	0.524
**Instances of Previous EEN Employment,** n (%)			0.156
<10	5 (26.3%)	9 (39.1%)	
10-20	5 (26.3%)	3 (13.0%)	
>20	4 (21.1%)	2 (8.7%)	
**Previous Patient EEN Treatment Experience,** n (%)			0.233
Yes	15 (78.9%)	17 (73.9%)	
No	4 (21.1%)	3 (13.0%)	
Unsure	0 (0%)	3 (13.0%)	

**Patients with Positive Previous EEN Experience,** n (%)<br>Yes<br>No<br>Mixed	 10 (52.6%)<br>0 (0%)<br>5 (26.3%)	 5 (21.7%)<br>3 (13.0%)<br>10 (43.5%)	**0.047**
**Frequency of Addressing Nutritional Issues,** n (%)<br>On every visit<br>On most visits<br>On some visits<br>Only per patient request	 8 (42.1%)<br>4 (21.1%)<br>7 (36.8%)<br>0 (0%)	 7 (30.4%)<br>14 (60.9%)<br>1 (4.3%)<br>1 (4.3%)	**0.013**
**Physician's Estimation of EEN Remission Rates,** n (%)<br>0-30%<br>30-60%<br>60-80%	 6 (31.6%)<br>6 (31.6%)<br>7 (36.8%)	 8 (34.8%)<br>11 (47.8%)<br>4 (17.4%)	0.331

EEN – exclusive enteral nutrition; IBD – inflammatory bowel diseases
