# Supplementary material for: Understanding Patient and Physician Perspectives on Exclusive Enteral Nutrition in Adults with Crohn’s Disease: Bridging the Gap in Nutritional Therapy
Source: Nutrients. 2025 Sep 12;17(18):2945. doi: 10.3390/nu17182945 (PMC12473139; doi:10.3390/nu17182945)
Supplement: Supplementary file 1 [file nutrients-17-02945-s001.zip › Table S6.pdf]

Table S6 - Multivariate Analysis of Variables that Affect Physician Willingness to Prescribe EEN

	Coefficient	Odds Ratio	95% CI	P-value
**Age**	0.032	1.03	0.952 - 1.121	0.430
**Female**	1.181	3.26	0.9 - 11.805	0.071
**Experience in Gastroenterology**	-0.017	0.982	0.909 - 1.063	0.661
**>15 Years IBD Care Experience**	0.121	1.129	0.296 - 4.312	0.858
**Treating >60 IBD Patients Per Year**	-0.300	0.740	0.197 - 2.787	0.650
**Israeli Medical School Graduate**	1.116	3.055	0.62 - 15.057	0.169
**Pediatric Gastro Collaboration**	-0.955	0.384	0.104 - 1.419	0.151
**Previous EEN Prescription**	1.540	4.666	1.115 - 19.538	**0.034**
**Addresses Nutrition During Most Visits**	0.207	1.2302	0.278 - 5.454	0.784

CI - confidence interval; EEN - exclusive enteral nutrition; IBD - inflammatory bowel disease
